# Supplementary material for: Visualizing Poloidal Orientation in DNA Minicircles
Source: bioRxiv. 2025 Aug 20:2025.08.20.671369. Preprint. [Version 1] doi: 10.1101/2025.08.20.671369 (PMC12393517; doi:10.1101/2025.08.20.671369)
Supplement: 1 [file NIHPP2025.08.20.671369V1-supplement-1.pdf]

bioRxiv preprint doi: https://doi.org/10.1101/2025.08.20.671369; this version posted August 20, 2025. The copyright holder for this preprint (which was not certified by peer review) is the author/funder, who has granted bioRxiv a license to display the preprint in perpetuity. It is made available under aCC-BY 4.0 International license.

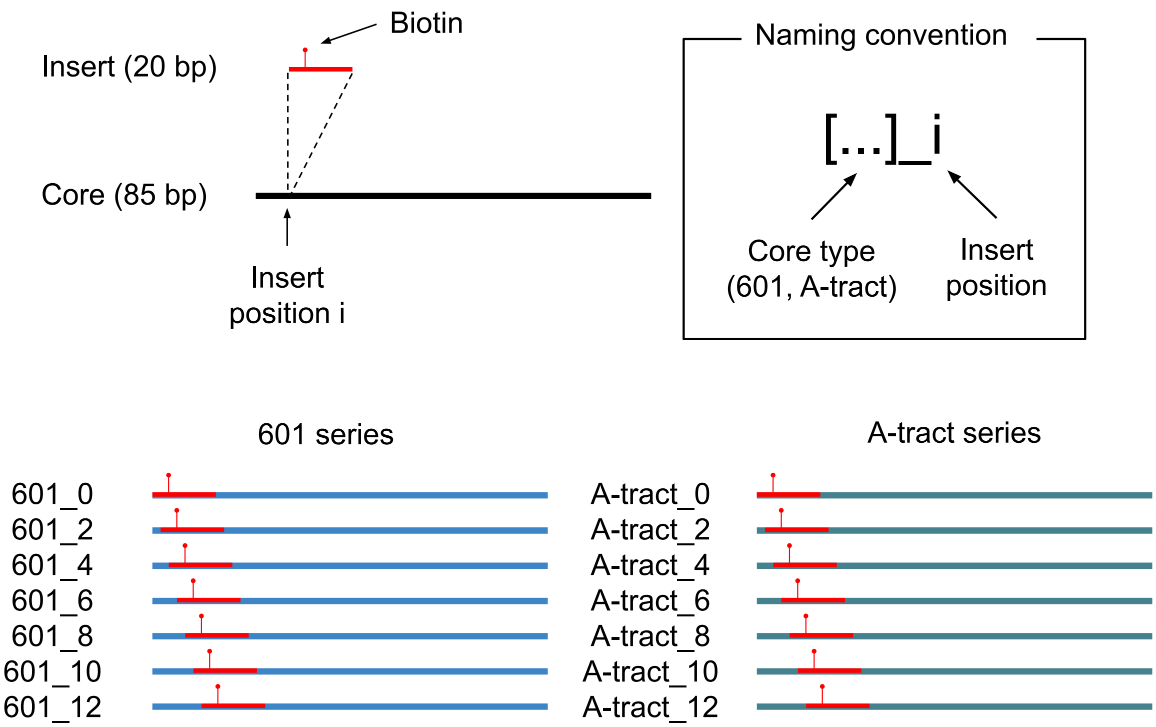

Figure S1. Sequences used for DNA minicircles in this study. The 85-nt core sequence was derived from either the left half of the 601 nucleosome positioning sequence (601 series) or from six in-phase A-tracts (A-tract series). A 20-nt segment containing a biotin-modified deoxythymidine was inserted at seven different positions. The sequences are named based on the core sequence and the insert position, and are listed in Table S1.

bioRxiv preprint doi: https://doi.org/10.1101/2025.08.20.671369; this version posted August 20, 2025. The copyright holder for this preprint (which was not certified by peer review) is the author/funder, who has granted bioRxiv a license to display the preprint in perpetuity. It is made available under aCC-BY 4.0 International license.

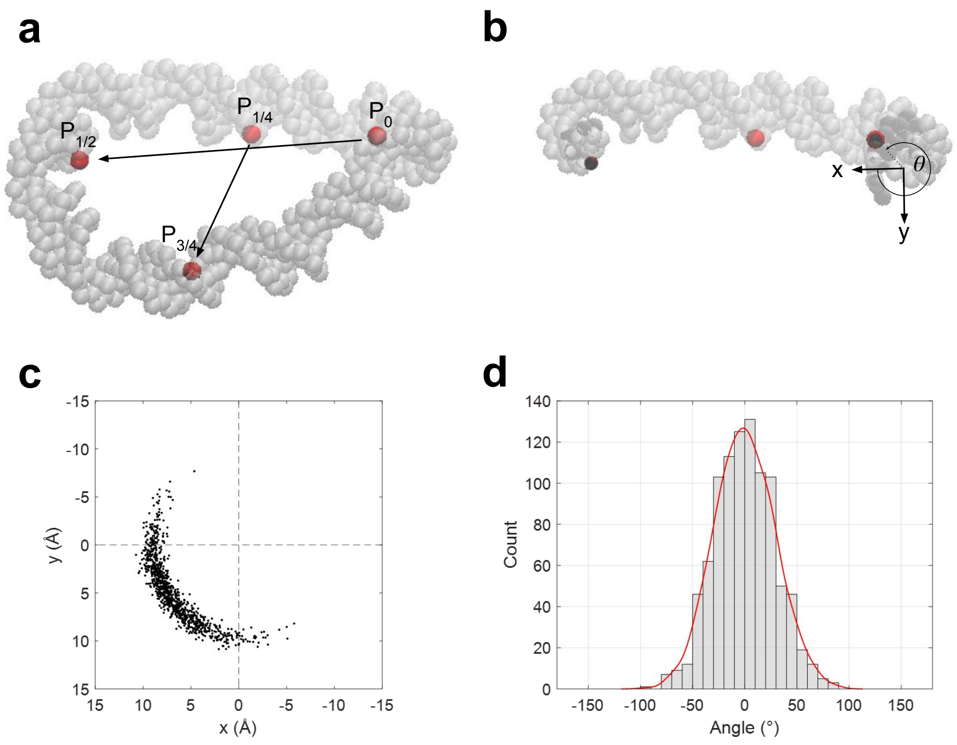

Figure S2. Poloidal angle calculation using 601_0 as an example. (a) A representative DNA minicircle conformation from MADna simulations. Four equidistant phosphate atoms are selected along the DNA backbone, including the phosphate (P_0) closest to biotin-dT. The arrow from P_0 to $P_{1/2}$ defines the x-axis, and the arrow from $P_{1/4}$ to $P_{3/4}$ defines the z-axis. The cross product of z and x yields the y-axis. (b) Clipped view of the same minicircle shown in (a). The xy-plane is defined as the plane containing P_0 and normal to the z-axis. The geometric center of mass of an 10-bp DNA segment centered at P_0 is computed, and its closest point on the xy-plane is designated as the origin O. The polar angle of P_0 with respect to this Cartesian coordinate system is defined as the poloidal angle (θ). (c) Positions of P_0 on the xy-plane from 950 minicircle conformations over a 95-ns simulation. The mean of the polar angles of these points is 35.9°, and the standard deviation (σ) is 28.7°. The points also vary in radial distance from the origin due to thermal fluctuations of the DNA helix. (d) A histogram of poloidal angles, calculated from the data shown in (c). For a better visualization, the distribution is translated to have zero mean. The gray bars represent the normalized histogram obtained from 950 conformations, and the red line represents the smoothed histogram obtained via kernel density estimation. FWHM of this bell-curve-like distribution is close to $2.35 \times \sigma \approx 67$ as expected for a normal distribution.

bioRxiv preprint doi: https://doi.org/10.1101/2025.08.20.671369; this version posted August 20, 2025. The copyright holder for this preprint (which was not certified by peer review) is the author/funder, who has granted bioRxiv a license to display the preprint in perpetuity. It is made available under aCC-BY 4.0 International license.

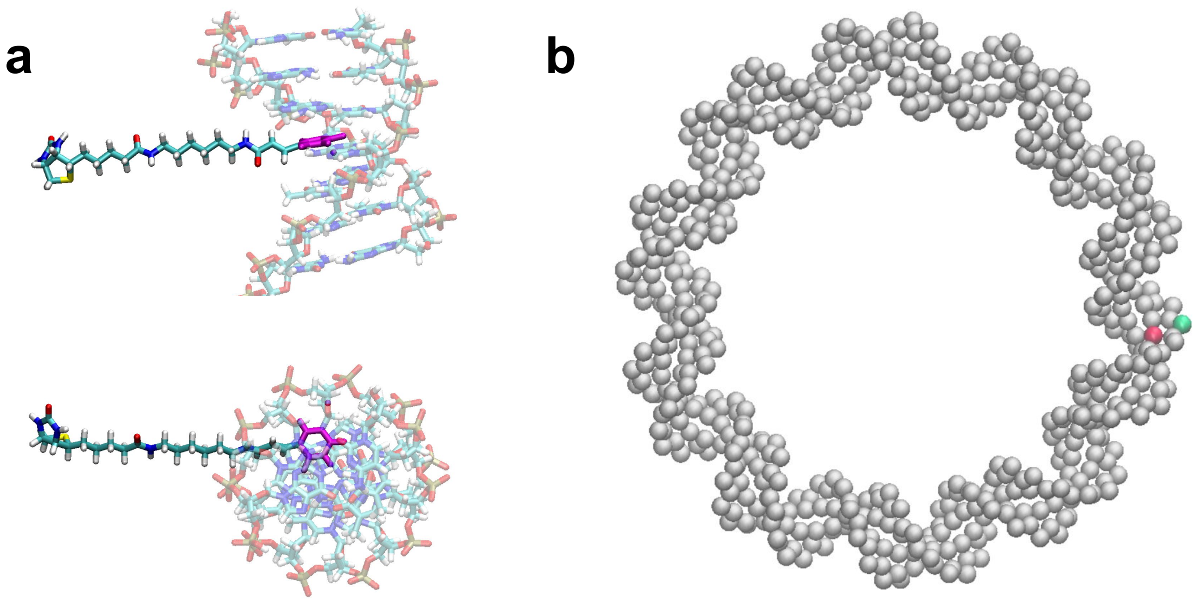

Figure S3. The position of the biotin group relative to DNA. (a) A atomistic-level structural model of the biotinylated DNA segment. This model features a 7-bp DNA duplex with a biotin-modified deoxythymidine (dT) at its center. The DNA duplex is rendered transparent to highlight the biotin-dT, with the thymine base colored magenta. Using MolView [47], the biotin moiety was constructed based on the chemical structure provided by Bioneer, and its 3D geometry was optimized. The biotin was then bonded to the dT, and the bond geometry further refined using Avogadro [48]. The top panel shows a side view, illustrating the biotin emerging from the major groove of the DNA helix, while the bottom panel shows the axial view. The biotin linker is approximately as long as the helix diameter, making the biotin accessible to NeutrAvidin. (b) Positions of phosphate and dT in a MADna-generated DNA minicircle. The red bead represents dT, and the green bead is the nearest phosphate to it. A biotin, if attached to the red bead, should emerge from the major groove, likely pointing out of the page, while the closest phosphate resides at the outermost position of the minicircle. Consequently, the poloidal angle of the phosphate is approximately 90° ahead of that of the biotin.

bioRxiv preprint doi: https://doi.org/10.1101/2025.08.20.671369; this version posted August 20, 2025. The copyright holder for this preprint (which was not certified by peer review) is the author/funder, who has granted bioRxiv a license to display the preprint in perpetuity. It is made available under aCC-BY 4.0 International license.

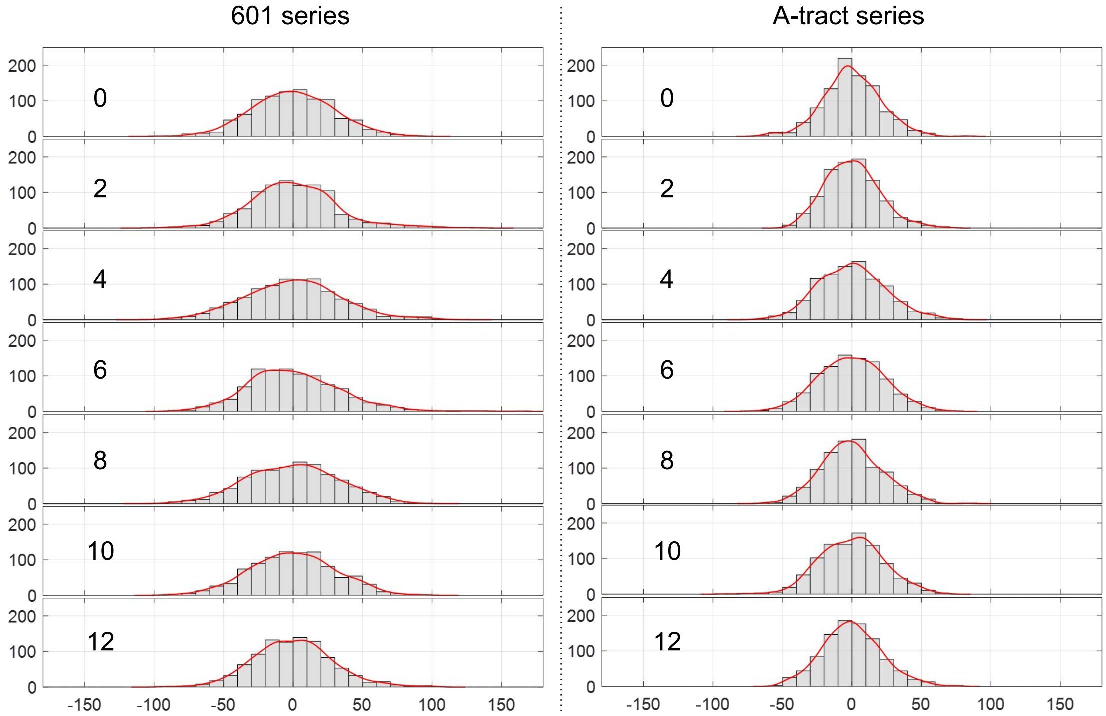

Figure S4. Histograms. The left and right columns show mean-centered poloidal angle histograms from the 601 series and from the A-tract series, respectively. From the top row to the bottom, the biotin-insert position changes from 0 to 12.

bioRxiv preprint doi: https://doi.org/10.1101/2025.08.20.671369; this version posted August 20, 2025. The copyright holder for this preprint (which was not certified by peer review) is the author/funder, who has granted bioRxiv a license to display the preprint in perpetuity. It is made available under aCC-BY 4.0 International license.

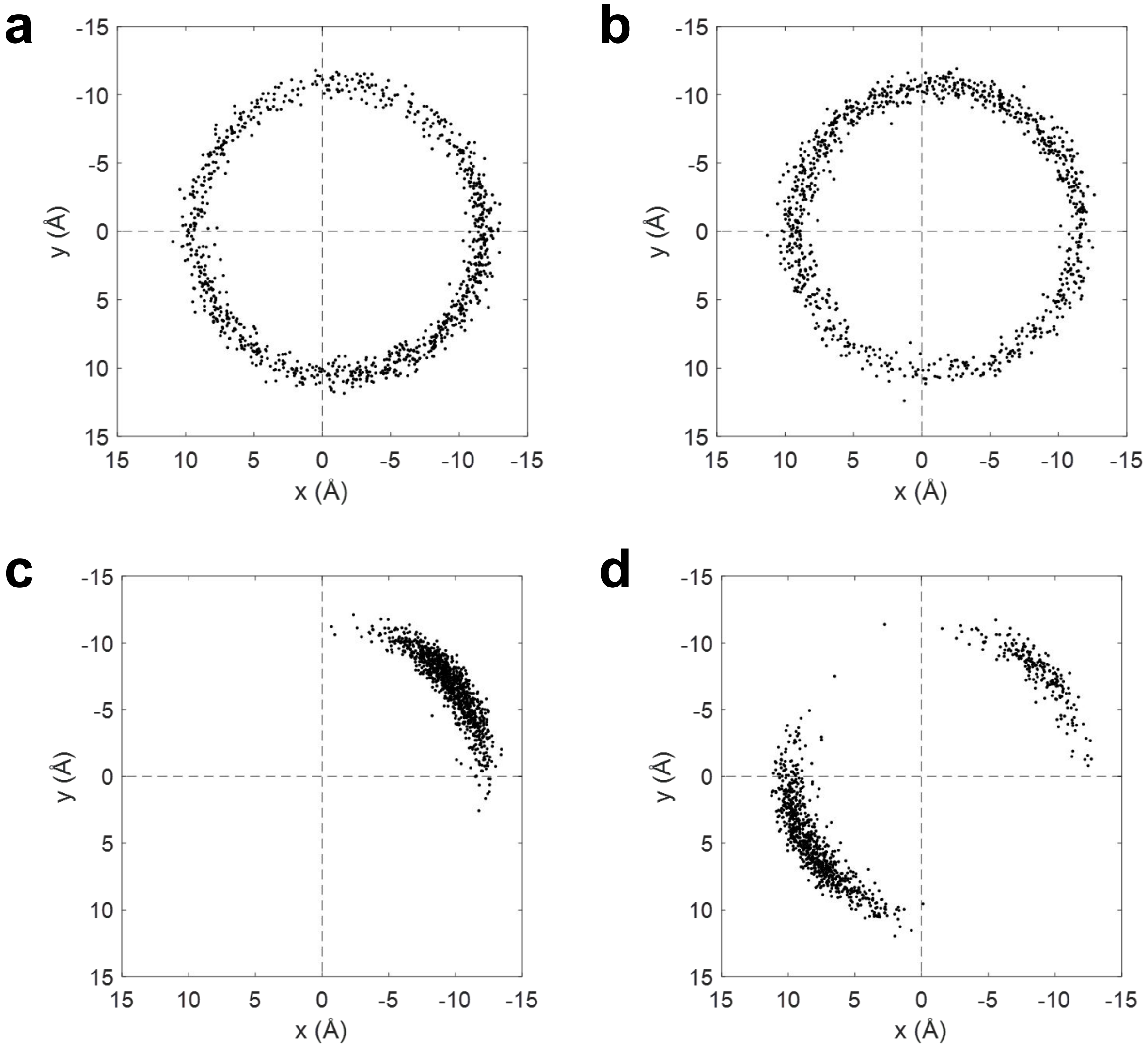

Figure S5. Poloidal angle distributions of various repeat sequences. All DNA minicircles are 88 bp in length with a linking number of 8, corresponding to a helical repeat of 11 bp. The horizontal and vertical axes follow the same definition as in Figure S2. Each dot represents the position of a phosphate atom relative to the centroid of its surrounding 10-bp DNA segment. (a) $\mathtt{C}_{88}$, a homopolymer of C. The geometric center of the distribution does not visibly align with the origin due to the bending of the 10-bp segment: an inward-facing phosphate atom is closer to the centroid than an outward-facing one. (b) $(\mathtt{TA})_{44}$, a TA dinucleotide repeat sequence (c) $(\mathtt{ACCCCCCCCCC})_8$ (d) $(\mathtt{ACCCCACCCCC})_8$

bioRxiv preprint doi: https://doi.org/10.1101/2025.08.20.671369; this version posted August 20, 2025. The copyright holder for this preprint (which was not certified by peer review) is the author/funder, who has granted bioRxiv a license to display the preprint in perpetuity. It is made available under aCC-BY 4.0 International license.

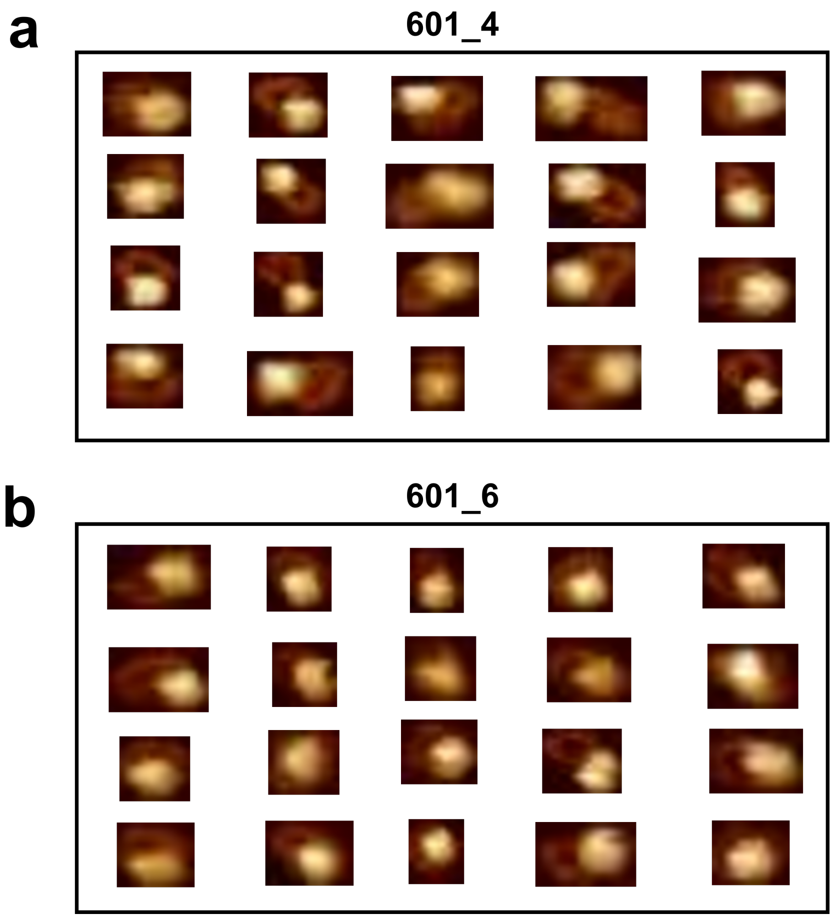

Figure S6. Examples of validated DNA-protein images from 601 series for minicircles with insert position 4 (a) and 6 (b).

bioRxiv preprint doi: https://doi.org/10.1101/2025.08.20.671369; this version posted August 20, 2025. The copyright holder for this preprint (which was not certified by peer review) is the author/funder, who has granted bioRxiv a license to display the preprint in perpetuity. It is made available under aCC-BY 4.0 International license.

	Sequence (5′ to 3′)
601_0	TCCGTCGAATATGATATCCTCGCGCTGTCCCCCGCGTTTTAGAATCCCGGTGCCGAGGCCGCTAAATTG<br>GTCGTAGACAGCTCTAGCACCGCTTAAACGCACGTA
601_2	TCCGTCGAATATGATATCCTTACGCGCTGTCCCCCGCGTTTTAGAATCCCGGTGCCGAGGCCGCTAAAT<br>TGGTCGTAGACAGCTCTAGCACCGCTTAAACGCACG
601_4	TCCGTCGAATATGATATCCTCGTACGCGCTGTCCCCCGCGTTTTAGAATCCCGGTGCCGAGGCCGCTAA<br>ATTGGTCGTAGACAGCTCTAGCACCGCTTAAACGCA
601_6	TCCGTCGAATATGATATCCTCACGTACGCGCTGTCCCCCGCGTTTTAGAATCCCGGTGCCGAGGCCGCT<br>AAATTGGTCGTAGACAGCTCTAGCACCGCTTAAACG
601_8	TCCGTCGAATATGATATCCTCGCACGTACGCGCTGTCCCCCGCGTTTTAGAATCCCGGTGCCGAGGCCG<br>CTAAATTGGTCGTAGACAGCTCTAGCACCGCTTAAA
601_10	TCCGTCGAATATGATATCCTAACGCACGTACGCGCTGTCCCCCGCGTTTTAGAATCCCGGTGCCGAGGC<br>CGCTAAATTGGTCGTAGACAGCTCTAGCACCGCTTA
601_12	TCCGTCGAATATGATATCCTTAAACGCACGTACGCGCTGTCCCCCGCGTTTTAGAATCCCGGTGCCGAG<br>GCCGCTAAATTGGTCGTAGACAGCTCTAGCACCGCT
A-tract_0	TCCGTCGAATATGATATCCTCGAAAAAACGGGCAAAAAACGGCAAAAAACGGGCAAAAAACGGCAAAAA<br>ACGGGCAAAAAATCTAGCACCGCTTAAACGCACGTA
A-tract_2	TCCGTCGAATATGATATCCTTACGAAAAAACGGGCAAAAAACGGCAAAAAACGGGCAAAAAACGGCAAA<br>AAACGGGCAAAAAATCTAGCACCGCTTAAACGCACG
A-tract_4	TCCGTCGAATATGATATCCTCGTACGAAAAAACGGGCAAAAAACGGCAAAAAACGGGCAAAAAACGGCA<br>AAAAACGGGCAAAAAATCTAGCACCGCTTAAACGCA
A-tract_6	TCCGTCGAATATGATATCCTCACGTACGAAAAAACGGGCAAAAAACGGCAAAAAACGGGCAAAAAACGG<br>CAAAAAACGGGCAAAAAATCTAGCACCGCTTAAACG
A-tract_8	TCCGTCGAATATGATATCCTCGCACGTACGAAAAAACGGGCAAAAAACGGCAAAAAACGGGCAAAAAAC<br>GGCAAAAAACGGGCAAAAAATCTAGCACCGCTTAAA
A-tract_10	TCCGTCGAATATGATATCCTAACGCACGTACGAAAAAACGGGCAAAAAACGGCAAAAAACGGGCAAAAA<br>ACGGCAAAAAACGGGCAAAAAATCTAGCACCGCTTA
A-tract_12	TCCGTCGAATATGATATCCTTAAACGCACGTACGAAAAAACGGGCAAAAAACGGCAAAAAACGGGCAAA<br>AAACGGCAAAAAACGGGCAAAAAATCTAGCACCGCT

bioRxiv preprint doi: https://doi.org/10.1101/2025.08.20.671369; this version posted August 20, 2025. The copyright holder for this preprint (which was not certified by peer review) is the author/funder, who has granted bioRxiv a license to display the preprint in perpetuity. It is made available under aCC-BY 4.0 International license.

Primers for making minicircles (5′ to 3′)	
Forward	[Phos]TCCGTCGAA[biotindT]ATGATATCCT
601_0 reverse	[Phos]TACGTGCGTTTAAGCGGT
601_2 reverse	[Phos]CGTGCGTTTAAGCGGTG
601_4 reverse	[Phos]TGCGTTTAAGCGGTGCTA
601_6 reverse	[Phos]CGTTTAAGCGGTGCTAGA
601_8 reverse	[Phos]TTTAAGCGGTGCTAGAGC
601_10 reverse	[Phos]TAAGCGGTGCTAGAGCTG
601_12 reverse	[Phos]AGCGGTGCTAGAGCTGTC
A-tract_0 reverse	[Phos]TACGTGCGTTTAAGCGGT
A-tract_2 reverse	[Phos]CGTGCGTTTAAGCGGTG
A-tract_4 reverse	[Phos]TGCGTTTAAGCGGTGCTA
A-tract_6 reverse	[Phos]CGTTTAAGCGGTGCTAGA
A-tract_8 reverse	[Phos]TTTAAGCGGTGCTAGATT
A-tract_10 reverse	[Phos]TAAGCGGTGCTAGATTTT
A-tract_12 reverse	[Phos]AGCGGTGCTAGATTTTTT

TABLE S1: List of DNA sequences and PCR primers for creating minicircles for AFM experiments.

bioRxiv preprint doi: https://doi.org/10.1101/2025.08.20.671369; this version posted August 20, 2025. The copyright holder for this preprint (which was not certified by peer review) is the author/funder, who has granted bioRxiv a license to display the preprint in perpetuity. It is made available under aCC-BY 4.0 International license.

Atom	r(Å)	θ_0(°)	z_0(Å)
S	8.7	0	0
B	4.2	5.1	-0.2
P	10.6	10.2	2.7
S2	8.7	102.4	-1.2
B2	4.0	93.6	-1.0
P2	10.6	123.8	-0.6

TABLE S2. Parameters used to construct DNA for MADna simulations.

bioRxiv preprint doi: https://doi.org/10.1101/2025.08.20.671369; this version posted August 20, 2025. The copyright holder for this preprint (which was not certified by peer review) is the author/funder, who has granted bioRxiv a license to display the preprint in perpetuity. It is made available under aCC-BY 4.0 International license.

**Name**	**Mean angle (°)**	**Std (°)**	**Mean x (Å)**	**Std (Å)**
601_0	35.9	28.7	6.5	2.7
601_2	93.7	30.7	-0.7	4.5
601_4	-160.9	34.1	-8.5	3.0
601_6	-99.9	32.5	-1.7	4.8
601_8	-19.7	32.4	7.3	2.2
601_10	25.6	30.3	7.1	2.3
601_12	78.5	28.2	1.5	4.1
A-tract_0	-171.1	20.6	-10.2	1.3
A-tract_2	-112.5	19.2	-3.9	3.2
A-tract_4	-45.7	23.8	5.9	2.5
A-tract_6	24.3	23.2	7.6	1.7
A-tract_8	88.7	21.9	0.1	3.5
A-tract_10	-176.9	22.7	-10.2	1.4
A-tract_12	-120.6	21.2	-5.2	3.4

TABLE S3. Mean poloidal angle and x position with standard deviations for each DNA minicircle.
